# Laparoscopic treatment of recurrent and chemoresistant cesarean scar choriocarcinoma

**DOI:** 10.1002/ccr3.3801

**Published:** 2021-01-18

**Authors:** Mehmet Sait Bakır, Özer Birge, Ceyda Karadag, Selen Doğan, Tayup Simsek

**Affiliations:** ^1^ Department of Gynecology Obstetrics Division of Gynecologic Oncology Akdeniz University Antalya Turkey

**Keywords:** cesarean scar, choriocarcinoma, laparoscopy, recurrent

## Abstract

Depending on the developing laparoscopic technique and experience, the treatment of cesarean scar choriocarcinoma can be safely performed laparoscopically by experts.

## Graphical abstract

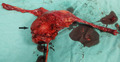

## 1 INTRODUCTION

We also aimed to present our experience about the diagnosis and treatment of our cesarean scar choriocarcinoma case whose tumor was chemoresistant and recurred in four months. The patient was referred to our clinic after the diagnosis of a choriocarcinoma. Patient's B‐HCG value was 15 600 mIU/mL, and pelvic MRI showed solid mass penetrating the serosa with a size of 3.5 × 2.9 cm in cesarean scar localization. Two months later after the end of chemotherapy, B‐HCG was found to be 586 mIU/mL and pelvic MRI reported a 30 × 27 × 28 mm mass lesion located in the anterior part of the caesarian scar line. Total laparoscopic hysterectomy and bilateral salpingectomy were performed. The B‐HCG value was 6.3 mIU/mL in the first‐week control of postoperative and <1 mIU/mL in the next week. Depending on the developing laparoscopic technique and experience, the treatment of cesarean scar choriocarcinoma can be safely performed laparoscopically by experts.

Gestational trophoblastic neoplasias (GTNs) are tumors that result from abnormal proliferation of trophoblastic tissues. These tumors can develop in relation to gestation, namely gestational choriocarcinoma or nongestational choriocarcinoma. Nongestational ones are choriocarcinomas that develop in the ovary. Gestational trophoblastic diseases are classified as complete or partial mole hydatiform, invasive mole, choriocarcinoma, placental site trophoblastic tumor (PSTT), and epithelioid trophoblastic tumors (ETTs).^1^

GTN is most commonly seen in the uterine corpus; due to increased cesarean rates in recent years, although it is quite rare, it can be seen in the cesarean scar line.^2^, ^3^, ^4^, ^5^, ^6^, ^7^, ^8^ Since GTNs are extremely chemosensitive, the rate of chemoresistance or relapse in Stage 1 patients is 2.9%, 8.3% for stage 2, 4.2% for stage 3 and 9.1% for stage 4.^9^ It has been emphasized that 71% of cesarean scar GTNs are chemoresistance and surgery is required.^8^

We also aimed to present our experience about the diagnosis and treatment of our cesarean scar choriocarcinoma case whose tumor was chemoresistant and recurred in four months.

## 2 CASE PRESENTATION

The patient is 29 years old with gravida 3, parity 2, and abortion 1. Her first pregnancy was completed in 2012 at term with cesarean section, and the second one was ended in 2017 at term with a cesarean section again. There was a history of revision curettage due to incomplete abortion in December 2018. The patient did not receive the pathology results after curettage and did not go to the postprocedure control examination. In May 2020, probe curettage was performed at another center because of the continuation of irregular vaginal bleeding. The patient was referred to our clinic after the diagnosis as a choriocarcinoma.

**FIGURE 1:**
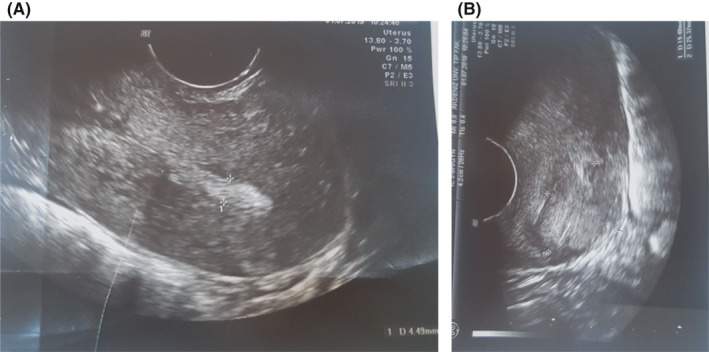
A, Normal endometrial cavity was seen in transvaginal ultrasonographic examination. B, On transvaginal ultrasonographic examination, 26 × 25 mm mass lesion was diagnosed in the old cesarean scar line of the retroverted uterus

**FIGURE 2:**
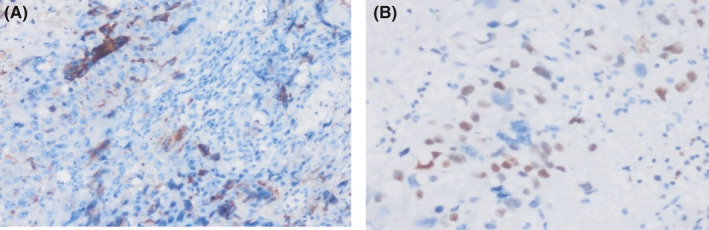
A, Beta‐HCG(x200): Tumor cells were positive with beta‐HCG administered by the immunohistochemical method. B, Ki‐67 (×400): High proliferation index was observed with Ki‐67 applied by the immunohistochemical method

**FIGURE 3:**
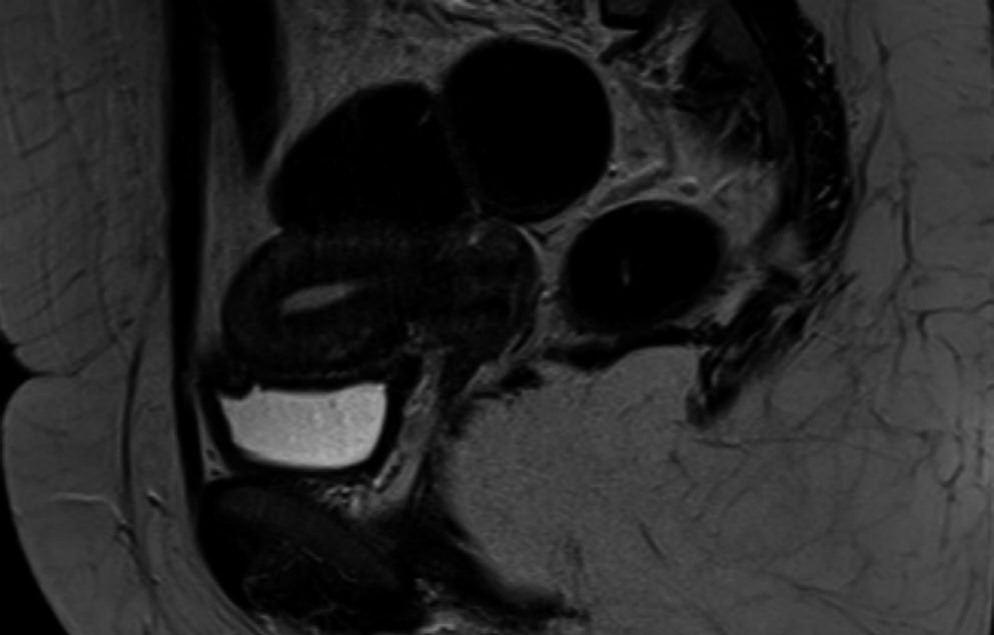
The tumoral mass is completely regressed in the cesarean scar line after chemotherapy

**FIGURE 4:**
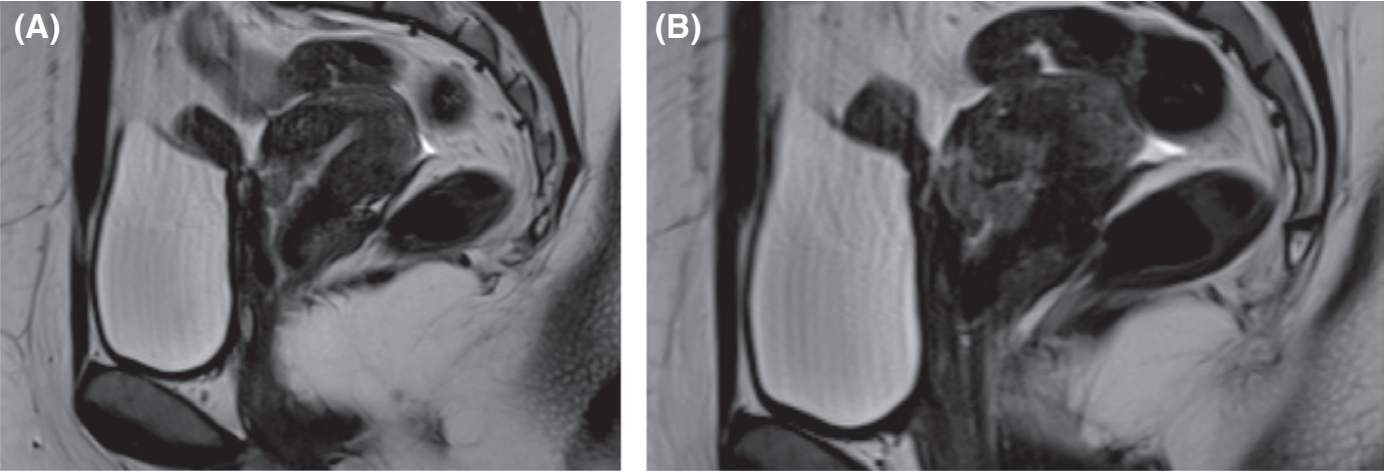
A and B, The appearance of a tumoral mass (recurrence) in the sagittal section of pelvic MRI on the CS scar line in the three months after chemotherapy

**FIGURE 5:**
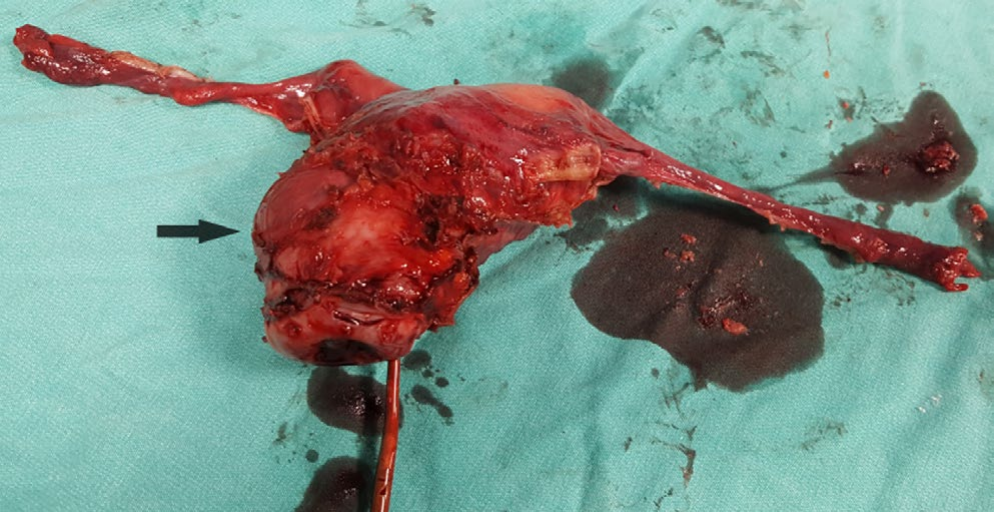
Postoperative view of hysterectomy material

During the gynecological examination of the patient, the perineum, vulva, and vagina were normal, there was minimal bleeding from cervix, the corpus uteri was normal in size, and the adnexa were normal. Transvaginal ultrasonography (TVS) revealed a 26x25 mm, irregular, solid lesion on the caesarian scar line, and it is extending to the uterine serosa (Figure 1 a, b). There was no sign in favor of gestational sac or molar pregnancy in the uterine cavity. Bilateral tuba and ovaries were normal. Patient's B‐HCG value was 15.600 mIU/ml. Abdominal and thorax CT, brain MRI, and pelvic MRI were performed for extrauterine spread. Liver and thyroid function tests were normal. Brain MRI and thorax CT findings were normal; pelvic MRI showed solid mass penetrating the serosa with a size of 3.5 × 2.9 cm in cesarean scar localization at uterus isthmus. The patient's pathology specimens were re‐examined by a gynecologic pathologist to confirm the diagnosis. B‐HCG in tumor cells immunohistochemically was positive, and high proliferation was observed with Ki‐67 (Figure 2A,B). Stage 1 and WHO score: 6 (1 point; occurrence after abortion, 2 points; index diagnosis at 7th month after pregnancy, 2 points; b‐HCG; being 15600, 1 point; tumor diameter measuring between 3‐4 cm) Single agent chemotherapy treatment was planned for the patient in the gynecological oncology council. After three‐cycle treatments of MTX + folinic acid and EMA‐CO [etoposide, methotrexate, actinomycin D cyclophosphate, and vincristine (Oncovin)], chemotherapy was started due to the plateau of B‐HCG. After 8 cycles of EMA‐CO chemotherapy, B‐HCG value is < 1 mIU/ml. At the same time, pelvic MRI showed that the mass on the cesarean scar line was completely regressed (Figure 3). Two months later after the end of chemotherapy, B‐HCG was found to be 77 mIU/mL and the patient suffered from vaginal bleeding. Ultrasound showed an irregular, solid mass with a diameter of 32 × 25 mm in the cesarean scar line, and a recurrence was considered. Pelvic MRI reported a 30 × 27 × 28 mm mass lesion located in the anterior part of the uterine isthmus (on the caesarian scar line), which restricting diffusion, showing heterogeneous contrast enhancement and containing necrotic areas (Figure 4A,B). Subsequent weekly B‐HCG values were 204 and 586 mIU/mL, respectively. Partial uterine resection or hysterectomy options were discussed with the patient due to future child request. The patient preferred laparoscopic hysterectomy and bilateral salpingectomy. In intraoperative exploration, it was observed that the cervico‐isthmic part was barrel‐shaped and extended toward parametrium bilaterally. The uterine serosa was thin in the isthmus. It was noticed that there was an increased vascularization above the bladder peritoneum. Peritoneal washing samples were taken. The retroperitoneal approach was decided due to technically difficult coagulation of the uterine artery because of the barrel‐shaped cervix and the presence of a bleeding‐prone tumor. By entering the retroperitoneal area from either side of the uterus, the ureters were visualized. Where uterine arteries originated from the hypogastric artery, they were coagulated with 5 mm ligasure. Total laparoscopic hysterectomy and bilateral salpingectomy were performed (Figure 5). Due to possible tumoral invasion, the hypervascularized bladder peritoneum was resected. Estimated blood loss was 10‐20 cc. The patient was discharged uncomplicated on the postoperative second day. The final pathology result was reported as choriocarcinoma. The B‐HCG value was 6.3 mIU/ml in the first‐week control of postoperative and < 1 mIU/ml in the next week. The patient who received weekly series of B‐HCG follow‐up was given two more cycles of EMA‐CO chemotherapy. The patient is well, without recurrence after three months.

## 3 DISCUSSION

Gestational trophoblastic neoplasias (GTNs) are tumors that result from abnormal proliferation of trophoblastic tissues after either molar pregnancy or nonmolar pregnancy and consist of invasive mole, choriocarcinoma, placental site trophoblastic tumor (PSTT), and epithelioid trophoblastic tumor (ETT).^1^ Today, women with GTN are treated with high cure rates, even at an advanced stage, because of the use of B‐HCG tests in follow‐up and highly effective chemotherapy regimens.^10^ Approximately 50% of GTNs occur after molar pregnancies, 25% after miscarriages, and the other 25% after term pregnancies.^11^ Approximately 15% invasive moles and 5% metastatic disease develop after complete mole hydatidiform; 1%‐4% invasive moles occur after partial mole, whereas metastatic disease does not develop.^10^ GTN developing after nonmolar pregnancy in Europe and America is 2‐7/100 000, whereas it is 5‐200/100 000 in Southern Asia and Japan.^12^ While the prevalence of GTNs after abortion is 1/15 000, the prevalence of GTNs after the term pregnancy is 1/150 000.^11^, ^12^ Choriocarcinoma develops most frequently after nonmolar pregnancy,^10^, ^11^, ^13^ and diagnosis is made later than those developed after molar pregnancy.^14^ When we look at our own patient, there is a history of abortion and the diagnosis was made after 7‐month delay.

Choriocarcinomas are the most aggressive tumors in GTNs, and they are characterized by early vascular and distant metastasis.^15^ Although choriocarcinomas are most frequently encountered in the uterine corpus, it was also seen in the ovary, fallopian tube, vagina, vulva, intestine, and cesarean scar.^2^, ^3^ The frequency of cesarean scar (CS) pregnancy, which is the rare form of ectopic pregnancy, has increased gradually due to the increase in cesarean rates in the world.^16^ The incidence of CS pregnancy is 1/2216 and constitutes 6.1% of all ectopic pregnancies.^17^

Cesarean scar choriocarcinoma is a very rare condition in the literature, and the first case report was published in 2014.^2^ As in our case, patients present with the complaint of irregular vaginal bleeding most frequently. In a different case report, the case was admitted with acute abdomen, resulting in a rupture of CS choriocarcinoma.^5^ Of cesarean scar pregnancies with catastrophic complications such as potential bleeding and rupture, early diagnosis and treatment are extremely important. It can easily be confused with cervical polyp, cervical cancer, and cesarean scar pregnancy; in diagnosis, CS choriocarcinoma may also be kept in mind of early diagnosis. A diagnosis of cesarean scar GTN can be made by transvaginal ultrasonography (TVS) by specialists or pelvic MRI.^3^ In our patient, intrauterine pregnancy and ectopic pregnancy were first excluded by transvaginal ultrasonography, but a solid irregular mass was observed in the cesarean scar line. The diagnosis was confirmed with MRI. According to the WHO prognostic score, our patient was admitted to stage 1 and low‐risk groups; single‐agent chemotherapy (MTX) was started. After 3 cycles, EMA‐CO was started because B‐HCG was drawing a plateau. After 8 cycles of EMA‐CO chemotherapy, the patient's B‐HCG was < 1 mIU/ml and the mass was completely regressed in pelvic MRI. However, in the next control of the patient, it was seen that the tumoral lesion reappeared in the cesarean scar line in our TVS and pelvic MR due to the increase in B‐HCG. Any metastasis was not detected in other regions. In a recent publication, 31 (3,3%) of 938 GTN patients had GTN in the cesarean scar, the majority of which were invasive mole cases. While choriocarcinoma was detected in 8 cases, the remaining cases were PSTT and ETT. In the same study, it was emphasized that 71% of 31 patients had to undergo hysterectomy.^8^ This may be due to poor blooding of the cesarean scar line, hiding the progenitor tumoral cells from chemotherapy. High‐dose chemotherapy autologous bone marrow transplantation or surgery option was considered for our patient who was accepted as resistant to chemotherapy. When the patient who did not have a child request accepted the surgery option, the operation was decided with minimally invasive approach. It was aimed to benefit from the advantages of laparoscopy such as early discharge, less postoperative pain, and earlier return to social life. Moreover, it has been shown that laparoscopic approach is safe and effective in CS pregnancies, especially in exophytic type.^18^ When we look at the literature, it is seen that most patients undergo hysterectomy by laparotomy.^2^, ^3^, ^5^, ^6^, ^7^, ^8^ Due to its highly vascular and bleeding‐prone tumors, in some of these cases, uterine artery embolization (UAE) was performed before the operation.^2^, ^3^, ^8^ In some centers, it was not routinely used due to the inability to process and some serious postoperative complications (labial and vaginal necrosis, vesicovaginal fistula, endometrial atrophy, and permanent amenorrhea).^19^ Since our clinic did not have a lot of UAE experience, uterine artery ligation was performed with laparoscopy. Occlusion of uterine arteries has several advantages. The first of these is as follows: since these tumors are prone to massive bleeding, it allows for safe partial resection by reducing uterine artery blood flow in patients with child expectation. Secondly, it prevents bleeding during dilatation curettage. As the third, a proactive intervention is made for postoperative intra‐abdominal bleeding. The operation was performed by our gynecological oncology team, and we noticed that the lower uterine segment, which is suitable for the characteristics of the tumor, was rather soft, barrel‐shaped, and dilated coarse vascularization in the bladder peritoneum. Bilateral uterine artery ligation was performed with a retroperitoneal approach in order to prevent the tumor rupture and bleeding. Total laparoscopic hysterectomy and bilateral salpingectomy were performed by working far from the tumoral area and acting meticulously.

Due to possible bladder peritoneal invasion, the peritoneal part with increased vascularization was removed. Intraoperative estimated blood loss was 10‐20 cc. Our patient, who was not drained, was discharged on the second postoperative day without any problem.

As a result, it should be kept in mind that cesarean scar choriocarcinoma is extremely rare, may be resistant to multiple chemotherapy, and surgery should be performed early. Depending on the developing laparoscopic technique and experience, the treatment of cesarean scar choriocarcinoma can be safely performed laparoscopically by experts.

## CONFLICT OF INTEREST

None declared.

## AUTHOR CONTRIBUTIONS

MSB and ÖB: wrote the manuscript. CK and SD: contributed to clinical follow‐up. TS: revised the manuscript.

## ETHICAL APPROVAL

None declared.

## INFORMED CONSENT

Informed consent was obtained from the patient to publish the case.
